# Morphology and phylogeny reveal two novel *Coryneum* species from China

**DOI:** 10.3897/mycokeys.56.35554

**Published:** 2019-07-10

**Authors:** Ning Jiang, Hermann Voglmayr, Cheng-Ming Tian

**Affiliations:** 1 The Key Laboratory for Silviculture and Conservation of the Ministry of Education, Beijing Forestry University, Beijing 100083, China Beijing Forestry University Beijing China; 2 Institute of Forest Entomology, Forest Pathology and Forest Protection, Department of Forest and Soil Sciences, BOKU-University of Natural Resources and Life Sciences, Franz Schwackhöfer Haus, Peter-Jordan-Straße 82/I, 1190 Vienna, Austria University of Natural Resources and Life Sciences Vienna Austria; 3 Division of Systematic and Evolutionary Botany, Department of Botany and Biodiversity Research, University of Vienna, Rennweg 14, A-1030 Vienna, Austria University of Vienna Vienna Austria

**Keywords:** *
Coryneaceae
*, *
Diaporthales
*, systematics, taxonomy

## Abstract

*Coryneum* is currently the sole genus of Coryneaceae in Diaporthales, distinguished from other diaporthalean genera by transversely distoseptate brown conidia. However, *Coryneum* species are presently difficult to identify because of variability and overlap of morphological characters and the lack of sequence data for most described species. During fungal collection trips in China, 13 *Coryneum* isolates were obtained from cankered branches of *Ilex* and *Quercus*. Morphological and phylogenetic analyses (ITS, LSU, *TEF1-α* and *RPB2*) revealed that these strains belong to two new species (*viz. Coryneumilicis***sp. nov.** and *C.songshanense***sp. nov.**), and three known species, *C.gigasporum*, *C.sinense*, and *C.suttonii*. *Coryneumilicis* has larger conidia and more distosepta than most *Coryneum* species. *Coryneumsongshanense* was similar to *C.sinense* from the same host genus, *Quercus*, in conidial length, but distinct in conidial width and by molecular data.

## Introduction

The genus *Coryneum* Nees is currently the only accepted genus in Coryneaceae and it forms a distinct phylogenetic lineage in Diaporthales (Senanayake et al. 2017, 2018, Voglmayr et al. 2017, Fan et al. 2018a, Jiang et al. 2018, Senwanna et al. 2018, Wijayawardene et al. 2017, 2018). The genus *Coryneum* was introduced based on the asexual morph, with *C.umbonatum* Nees as the type species (Nees von Esenbeck 1816), and the sexual morph *Pseudovalsa* Ces. & De Not. was introduced later, based on *P.lanciformis* (Fr.) Ces. & De Not. (Cesati & De Notaris 1863). *Coryneum* was recommended to be adopted due to priority and the need of fewer new combinations (Rossman et al. 2015).

Most *Coryneum* species were considered as phytopathogens, which were discovered from cankers and dieback of shoots and twigs (Wijayawardene et al. 2016, Senanayake et al. 2017, Jiang et al. 2018). However, diseases are commonly mild and only rarely cause serious symptoms in the hosts. Additionally, pathogenicity tests have not yet been conducted.

*Coryneum* species are generally considered highly host-specific, and 28 species and a variety were accepted in this genus before this study (Sutton 1975, 1980, Wijayawardene et al. 2016, Jiang et al. 2018, Senwanna et al. 2018). *Coryneumterrophilum* was the only species isolated from soil, and the others were reported from dead branches (Table 1). Fagales species are the major hosts of *Coryneum* species, and host trees from other orders are also hardwoods with rough barks (Table 1).

**Table 1.** Hosts, conidial sizes, and numbers of distosepta of currently accepted *Coryneum* species.

Species	Host genus	Host family	Host order	Conidial size (μm)	No. of distosepta	References
* C. arausiacum *	* Quercus *	Fagaceae	Fagales	42–56 × 13–16	4–5	Senanayake et al. (2017)
* C. betulinum *	* Betula *	Betulaceae	Fagales	31–36 × 14–17	4–5	Sutton (1975)
* C. calophylli *	* Calophyllum *	Guttiferae	Parietales	38–48 × 12.5–14.5	5–6	Sutton (1975)
* C. carpinicola *	* Carpinus *	Betulaceae	Fagales	50–68 × 8–11	7–11	Sutton (1975)
* C. castaneicola *	* Castanea *	Fagaceae	Fagales	56–80 × 9.5–13	5–8	Sutton (1975)
* C. cesatii *	* Aesculus *	Hippocastanaceae	Sapindales	80–90 × 13–15	6–7	Sutton (1975)
* C. clusiae *	* Clusia *	Clusiaceae	Malpighiales	30–40 × 20–30	3–5	Sutton (1975)
* C. compactum *	* Ulmus *	Ulmaceae	Urticales	40–58 × 15–21	4–6	Sutton (1975)
* C. depressum *	* Quercus *	Fagaceae	Fagales	44–53 × 19–23	4–6	Sutton (1975)
* C. elevatum *	* Quercus *	Fagaceae	Fagales	56–69 × 24–28	5–7	Sutton (1975)
* C. gigasporum *	* Castanea *	Fagaceae	Fagales	88–117 × 18–23	7–9	Jiang et al. (2018)
* C. gregoryi *	* Eucalyptus *	Myrtaceae	Myrtales	32.5–43 × 12–16	5–9	Sutton and Sharma (1983)
* C. heveanum *	* Hevea *	Euphorbiaceae	Malpighiales	40–68 × 14–20	4–6	Senwanna et al. (2018)
* C. ilicis *	* Ilex *	Aquifoliaceae	Sapindales	82–105 × 9.5–12.5	10–11	This study
* C. japonicum *	* Quercus *	Fagaceae	Fagales	45–60 × 11–12	5–7	Sutton (1975)
* C. lanciforme *	* Betula *	Betulaceae	Fagales	45–53 × 16–18	4–6	Sutton (1975)
* C. megaspermum *	* Quercus *	Fagaceae	Fagales	73–97 × 13–16	7–11	Sutton (1980)
C. megaspermum var. cylindricum	* Quercus *	Fagaceae	Fagales	100–125 × 10–13	7–8	Sutton (1975)
* C. modonium *	* Castanea *	Fagaceae	Fagales	50–71 × 14–19	5–8	Sutton (1975)
* C. neesii *	* Quercus *	Fagaceae	Fagales	68–82 × 18–22	6–8	Sutton (1975)
* C. pruni *	* Prunus *	Rosaceae	Rosales	14–23 × 5.5–9	4–5	Wijayawardene et al. (2016)
* C. psidii *	* Psidium *	Myrtaceae	Myrtales	25–40 × 14–17	5–6	Sutton (1975)
* C. pyricola *	* Pyrus *	Rosaceae	Rosales	61–70 × 24–32	5–7	Sutton (1975)
* C. quercinum *	* Quercus *	Fagaceae	Fagales	45–60 × 14–16	6–7	Muthumary and Sutton (1986)
* C. sinense *	* Quercus *	Fagaceae	Fagales	50–76 × 13–17	5–7	Jiang et al. (2018)
* C. songshanense *	* Quercus *	Fagaceae	Fagales	51–76 × 9–11.5	5–7	This study
* C. stromatoideum *	* Tsuga *	Pinaceae	Pinales	105–180 × 16–20	9–17	Sutton (1975)
* C. suttonii *	* Castanea *	Fagaceae	Fagales	60–76 × 10–14.5	4–5	Jiang et al. (2018)
* C. sydowianum *	* Alnus *	Betulaceae	Fagales	50–58 × 14–17	5–6	Sutton (1975)
* C. terrophilum *	NA	NA	NA	25–55 × 15–24	3–7	Sutton and Sharma (1983)
* C. umbonatum *	* Quercus *	Fagaceae	Fagales	57–72 × 13–16	5–7	Sutton (1975)

Molecular phylogenies based on multi-gene loci including the internal transcribed spacer (ITS) and the large subunit (LSU) regions of the nuclear rDNA, translation elongation factor-1α (*TEF1-α*) and the second largest subunit of the RNA polymerase II (*RPB2*) have been widely used to infer species delimitation within many genera in Diaporthales (Voglmayr et al 2012, 2017, 2019, Voglmayr and Jaklitsch 2014, Fan et al. 2018b, Jiang et al. 2019), and are particularly important in speciose genera like *Coryneum*. Hence, DNA extraction from known species and fresh collections from the potential hosts will greatly improve the elucidation of species concept and circumscription in *Coryneum*. Thus, the main objectives of the present study were to identify *Coryneum* taxa based on morphology and phylogenetic evidence, and to analyse the relationships between *Coryneum* species and host genera.

## Materials and methods

### Sample collection and isolation

Sample collection trips were conducted in Beijing, Hebei and Shaanxi Provinces of China during June to October in 2017 and 2018, aiming to collect fresh specimens with *Coryneum*–like taxa. Fagales plants were the main hosts and other hardwoods with rough barks were also investigated. Healthy branches and twigs were covered by green leaves, hence the dying and dead materials were conspicuous during our investigations. Asexual fruiting bodies were easily discovered as black spots on the host barks. Tree tissues with fruiting bodies were cut into small pieces, packed in paper bags and taken to the laboratory for further studies. Isolations were obtained by removing the ascospores or conidial masses from the fruiting bodies on to clean potato dextrose agar (PDA) plates, which were incubated at 25 °C until spores germinated. Single germinating spores were transferred on to new PDA plates, which were kept at 25 °C in the dark. Specimens were deposited at the Museum of the Beijing Forestry University (BJFC) and axenic cultures are maintained at the China Forestry Culture Collection Centre (CFCC).

### Morphological analysis

Species identification was based on the morphological characters of the sexual and asexual morphs produced on natural substrates. Cross-sections were prepared manually using a double-edged blade under a Leica stereomicroscope (M205 FA). Photomicrographs were captured with a Nikon Eclipse 80i microscope equipped with a Nikon digital sight DS-Ri2 high-definition colour camera, using differential interference contrast (DIC) illumination and the Nikon software, NIS-Elements D Package 3.00. Measurements of ascospores and conidia are reported as the maximum and minimum in parentheses and the range representing the mean ± standard deviation of the number of measurements is given in parentheses (Voglmayr et al. 2017). Cultural characteristics of isolates incubated on MEA in the dark at 25 °C were recorded.

Recognition and identification of *Coryneum* species were based on fruiting bodies formed on tree bark, supplied by conidiomata produced on PDA plates. Ascomata and conidiomata from tree bark were sectioned by hand using a double-edged blade, and conidiomata from PDA plates were picked using a needle, which were observed under a dissecting microscope. At least 10 conidiomata/ascomata, 10 asci, and 50 conidia/ascospores were measured to calculate the mean sizes and standard deviation. Microscopy photographs were captured with a Nikon Eclipse 80i compound microscope equipped with a Nikon digital sight DS-Ri2 high definition colour camera, using differential interference contrast illumination.

### DNA extraction, PCR amplification and sequencing

Genomic DNA was extracted from colonies grown on cellophane-covered PDA plates using a modified CTAB method (Doyle and Doyle 1990). PCR amplifications were performed in a DNA Engine Peltier Thermal Cycler (PTC-200; Bio-Rad Laboratories, Hercules, CA, USA). The primer sets ITS1/ITS4 (White et al. 1990) were used to amplify the ITS region. The primer pair LR0R/LR5 (Vilgalys and Hester 1990) was used to amplify the LSU region. The primer pairs EF1-688F/EF1-986R or EF1-728F/TEF1-LLErev (Carbone and Kohn 1999, Jaklitsch et al. 2006, Alves et al. 2008) were used to amplify *TEF1-α* gene. The primer pair dRPB2-5f/dRPB2-7r (Voglmayr et al. 2016) was used to amplify the *RPB2* gene. The polymerase chain reaction (PCR) assay was conducted as described by Fan et al. (2018a). PCR amplification products were assayed via electrophoresis in 2 % agarose gels. DNA sequencing was performed using an ABI PRISM® 3730XL DNA Analyzer with a BigDye Terminater Kit v.3.1 (Invitrogen, USA) at the Shanghai Invitrogen Biological Technology Company Limited (Beijing, China). Novel sequences generated in the current study were deposited in GenBank (Table 2).

**Table 2.** Strains used in the phylogenetic tree and their culture accession and GenBank numbers. Strains from this study are in bold.

Species	Strains	GenBank numbers			
		ITS	LSU	TEF1-*α*	RPB2
* Coryneum castaneicola *	CFCC 52315	MH683551	MH683559	MH685731	MH685723
* Coryneum castaneicola *	CFCC 52316	MH683552	MH683560	MH685732	MH685724
* Coryneum depressum *	D202	MH674330	MH674330	MH674338	MH674334
* Coryneum heveanum *	MFLUCC 17-0369	MH778707	MH778703	MH780881	NA
* Coryneum heveanum *	MFLUCC 17-0376	MH778708	MH778704	NA	NA
* Coryneum gigasporum *	CFCC 52319	MH683557	MH683565	MH685737	MH685729
* Coryneum gigasporum *	CFCC 52320	MH683558	MH683566	MH685738	MH685730
*** Coryneum gigasporum ***	**G14**	**MK799957**	**MK799944**	**MK799830**	**MK799820**
*** Coryneum gigasporum ***	**G15**	**MK799958**	**MK799945**	**MK799831**	**MK799821**
*** Coryneum ilicis ***	**CFCC 52994**	**MK799948**	**MK799935**	**NA**	**NA**
*** Coryneum ilicis ***	**CFCC 52995**	**MK799949**	**MK799936**	**NA**	**NA**
*** Coryneum ilicis ***	**CFCC 52996**	**MK799950**	**MK799937**	**NA**	**NA**
* Coryneum lanciforme *	D215	MH674332	MH674332	MH674340	MH674336
* Coryneum modonium *	D203	MH674331	MH674331	MH674339	MH674335
* Coryneum modonium *	CBS 130.25	MH854812	MH866313	NA	NA
* Coryneum sinense *	CFCC 52452	MH683553	MH683561	MH685733	MH685725
* Coryneum sinense *	CFCC 52453	MH683554	MH683562	MH685734	MH685726
*** Coryneum sinense ***	**X20**	**MK799952**	**MK799939**	**MK799825**	**MK799815**
*** Coryneum sinense ***	**X23**	**MK799953**	**MK799940**	**MK799826**	**MK799816**
*** Coryneum sinense ***	**X60**	**MK799951**	**MK799938**	**MK799824**	**MK799814**
*** Coryneum songshanense ***	**CFCC 52997**	**MK799946**	**MK799933**	**MK799822**	**MK799812**
*** Coryneum songshanense ***	**CFCC 52998**	**MK799947**	**MK799934**	**MK799823**	**MK799813**
* Coryneum suttonii *	CFCC 52317	MH683555	MH683563	MH685735	MH685727
* Coryneum suttonii *	CFCC 52318	MH683556	MH683564	MH685736	MH685728
*** Coryneum suttonii ***	**Z15-1**	**MK799954**	**MK799941**	**MK799827**	**MK799817**
*** Coryneum suttonii ***	**Z17**	**MK799955**	**MK799942**	**MK799828**	**MK799818**
*** Coryneum suttonii ***	**Z86**	**MK799956**	**MK799943**	**MK799829**	**MK799819**
* Coryneum umbonatum *	D201	MH674329	MH674329	MH674337	MH674333

### Phylogenetic analyses

Sequences generated from the above primers of the different genomic regions (ITS, LSU, *TEF1-α* and *RPB2*) were analysed in comparison to known species, *Stilbosporamacrosperma* (CBS 115073) and *Stegonsporiumpyriforme* (CBS 120522) were used as the outgroup taxa (Jiang et al. 2018). All sequences were aligned using MAFFT v. 6 (Katoh and Toh 2010) and edited manually using MEGA v. 6 (Tamura et al. 2013). Phylogenetic analyses were performed using PAUP v. 4.0b10 for maximum parsimony (MP) analysis (Swofford 2003), and PhyML v. 3.0 for Maximum Likelihood (ML) analysis (Guindon et al. 2010).

A partition homogeneity test with heuristic search and 1000 replicates was performed using PAUP v. 4.0b10 to assess incongruence among the ITS, LSU, *TEF1-α*, and *RPB2* sequence datasets in reconstructing phylogenetic trees. MP analysis was run using a heuristic search option of 1000 search replicates with random-addition of sequences with a tree bisection and reconnection (TBR) algorithm; branches of zero length were collapsed (collapse = minbrlen), and all equally most parsimonious trees were saved. Other calculated parsimony scores were tree length (TL), consistency index (CI), retention index (RI), and rescaled consistency (RC). ML analysis was performed using a GTR site substitution model, including a gamma-distributed rate heterogeneity and a proportion of invariant sites (Guindon et al. 2010). The branch support was evaluated using a bootstrapping method of 1000 bootstrap replicates (Hillis and Bull 1993). The MP bootstrap analyses were done with the same settings as for the heuristic search, but with 10 rounds of heuristic search during each bootstrap replicate. Phylograms were shown using FigTree v. 1.4.3 (Rambaut 2016).

## Results

### Phylogenetic analyses

**Figure 1.**
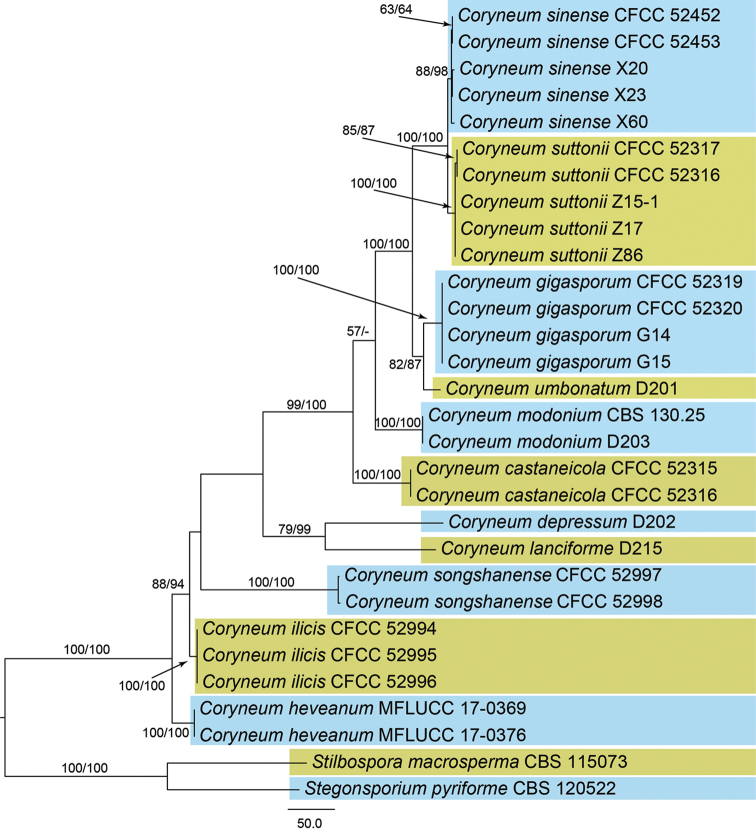
Phylogenetic tree based on an MP analysis of a combined DNA dataset of ITS, LSU, *TEF1-α* and *RPB2* gene sequences for the species of *Coryneum*. Bootstrap values ≥ 50 % for MP/ML analyses are presented at the branches. Scale bar = 50 nucleotide substitutions.

The alignment based on the combined sequence dataset (ITS, LSU, *TEF1-α*, and *RPB2*) included 30 ingroup taxa and two outgroup taxa (*Stilbosporamacrosperma* and *Stegonsporiumpyriforme*), comprising 3544 characters in the aligned matrix. Of these, 2570 characters were constant, 267 variable characters were parsimony-uninformative and 706 characters were parsimony informative. The partition homogeneity test resulted in an insignificant value (level 95%), indicating that ITS, LSU, *TEF1-α* and *RPB2* sequence dataset could be combined. The MP analysis resulted in 2 equally most parsimonious trees; the first tree (TL = 1624, CI = 0.784, RI = 0.822, RC = 0.645) is shown in Fig. 1. The two MP trees were identical, except for an interchanged position of *C.ilicis* and *C.songshanense* (not shown). Tree topology of the best tree revealed by the ML analyses was identical to that of the MP tree shown. The phylogram based on the four gene sequences showed that the accessions here studied represented 2 new and 3 known species in *Coryneum* (Fig. 1).

### Taxonomy

#### Coryneum ilicis

Taxon classificationFungiDiaporthalesPseudovalsaceae

C.M. Tian & N. Jiang sp. nov.

830201

Figure 2

##### Diagnosis.

*Coryneumilicis* is characterised by its host, *Ilexpernyi*, and large conidia with 10–11 distosepta.

##### Holotype.

CHINA. Shaanxi Province: Zhashui County, on branches of *Ilexpernyi*, 12 August 2017, N. Jiang (holotype: BJFC-S1720; ex-type culture from ascospore: CFCC 52994; living culture from conidium: CFCC 52996).

##### Etymology.

Named after the host genus on which it was collected, *Ilex*.

##### Description.

Associated with canker on branches of *Ilexpernyi*. ***Sexual morph***: Pseudostromata 0.5–1.5 mm diam., typically distinct, circular, without perithecial bumps, containing 1 or 2 perithecia embedded in a well-developed entostroma. Central column and entostroma grey. Ostioles inconspicuous and often invisible at the surface of the ectostromatic disc. Perithecia (350–)500–700(–850) μm diam. (n = 20), globular, somewhat flattened at the base. Asci 110–155 × 13–20 μm, 8-spored, unitunicate, clavate, shortly pedicellate, apically rounded, with a conspicuous apical ring. Ascospores (26.2–)29.7–35.5(–36.2) × (11.0–)11.8–14.3(–15.2) μm, l/w = (1.9–)2.2–2.9(–3.2) (n = 50), 1-seriate, fusiform, ends pointed, uniseptate, constricted at the septa, hyaline, guttulate, smooth-walled. ***Asexual morph***: Conidiomata acervular, 0.2–1 mm wide, 0.2–1.2 mm high, solitary, erumpent through the outer periderm layers of the host, scattered, surface tissues above slightly domed. Conidiophores 40–85 μm long, 3–7 μm wide, branched, cylindrical, septate, hyaline at the apex, pale brown at the base. Conidiogenous cells holoblastic, integrated, indeterminate, cylindrical, expanding towards the apices, pale brown, smooth, with 0–1 percurrent extensions. Conidia (82–)87–95(–105) × (9.5–)10.5–11.5(–12.5) μm, l/w = (7.4–)7.7–9.1(–9.3) (n = 50), variable in shape, curved, broadly fusiform to fusiform, cylindrical or clavate, dark brown, smooth-walled, 10–11-distoseptate, apical cell with a hyaline tip, truncate and black at the base.

**Figure 2.**
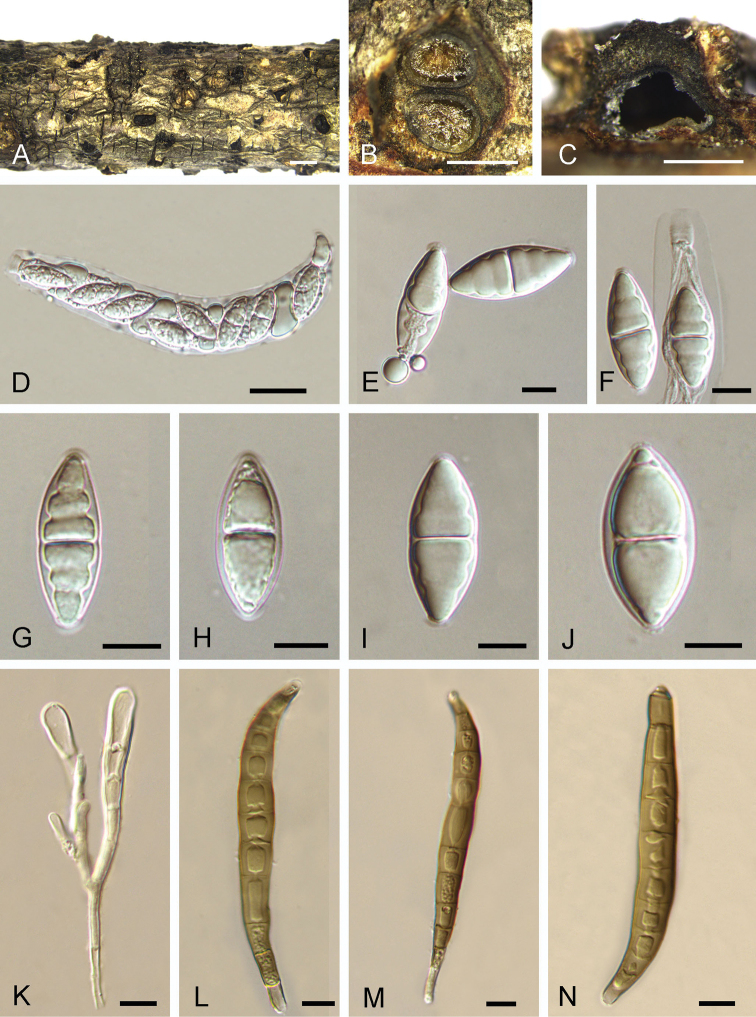
*Coryneumilex* from *Ilexpernyi* (BJFC-S1720, holotype) **A** Fruiting bodies on natural substrate in surface view **B** pseudostroma in transverse section, showing perithecia and gray entostroma **C** longitudinal sections through pseudostromata **D** ascus **E–J** ascospores **K** conidiophores **L–N** conidia. Scale bars: 1 mm (**A**); 0.5 mm (**B, C**); 20 μm (**D**); 10 μm (**E–N**).

##### Culture characters.

On PDA at 25 °C, colonies growing slowly and unevenly, reaching 70 mm diam. within 25 d, gradually becoming brownish dark grey in colour with scant cottony aerial mycelium, asexual morphs developed after 35 d.

##### Additional specimen examined.

CHINA. Shaanxi Province: Zhashui County, on branches of *Ilexpernyi*, 12 August 2017, N. Jiang (isotype: BJFC-S1721; living culture: CFCC 52995).

##### Notes.

*Coryneumilicis* is the sole species known from the host genus *Ilex*; it can be easily recognised by host association and phylogeny (Fig. 1). Morphologically, conidia of *Coryneumilicis* are larger and have more distosepta than in most of the other species (Table 1).

#### Coryneum songshanense

Taxon classificationFungiDiaporthalesPseudovalsaceae

C.M. Tian & N. Jiang sp. nov.

830202

Figure 3

##### Diagnosis.

*Coryneumsongshanense* can be distinguished from the morphologically similar *C.sinense* by its narrower conidia.

##### Holotype.

CHINA. Beijing City: Songshan Mountain, on dead twigs of *Quercusdentata*, 15 June 2018, N. Jiang & C.M. Tian (holotype: BJFC-S1722; ex-type culture from ascospore: CFCC 52997).

##### Etymology.

Named after the mountain on which it was collected, Songshan Mountain.

##### Description.

Associated with canker on twigs of *Quercusdentata*. ***Sexual morph***: Pseudostromata 0.3–1 mm diam., typically distinct, circular, without perithecial bumps, containing up to 6 perithecia embedded in a well-developed entostroma. Ectostromatic disc distinct, circular, black, 0.3–0.5 mm diam. Central column and entostroma grey. Ostioles inconspicuous and often invisible at the surface of the ectostromatic disc. Perithecia (150–)200–450(–550) μm diam. (n = 20), globular, somewhat flattened at the base with black short neck. Asci 75–145 × 17–23 μm, 8-spored, unitunicate, clavate, shortly pedicellate, apically rounded, with an inconspicuous apical ring. Ascospores (24.1–)25.5–35.4(–38.2) × (7.5–)7.9–9.8(–10.6) μm, l/w = (3.0–)3.3–3.8(–4.2) (n = 50), 2-seriate, fusiform, ends pointed, uniseptate or aseptate, not constricted at the septa, hyaline, guttulate, smooth-walled. ***Asexual morph***: Conidiomata acervular, 0.2–0.6 mm wide, 0.2–0.5 mm high, solitary, erumpent through the outer periderm layers of the host, scattered, surface tissues above slightly domed. Conidiophores 15–35 μm long, 4–7 μm wide, unbranched, cylindrical, septate, hyaline at the apex, pale brown at the base. Conidiogenous cells holoblastic, integrated, indeterminate, cylindrical, expanding towards the apices, pale brown, smooth, with 0–1 percurrent extensions. Conidia (51–)56–67(–76) × (9–)10–11(–11.5) μm, l/w = (5.2–)5.5–6.9(–8.1) (n = 50), variable in shape, curved, broadly fusiform to fusiform, cylindrical or clavate, dark brown, smooth-walled, 5–7-distoseptate, apical cell with a hyaline tip, truncate and black at the base.

**Figure 3.**
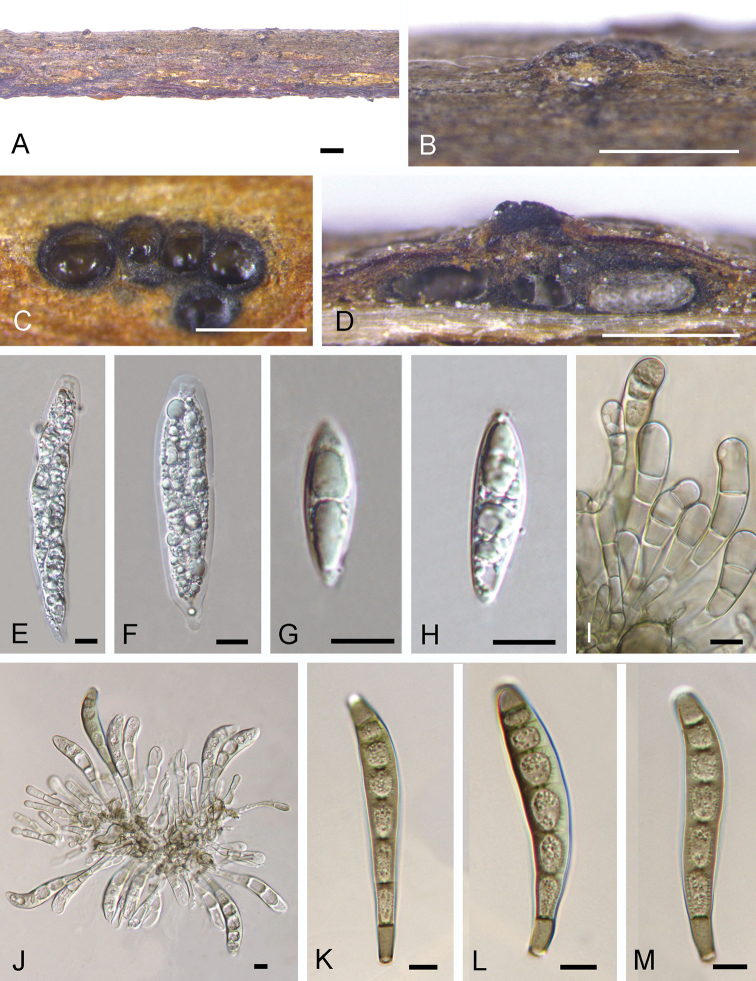
*Coryneumsongshanense* from *Quercusdentata* (BJFC-S1722, holotype) **A, B** Fruiting bodies on natural substrate in surface view **C** pseudostroma in transverse section, showing perithecia and gray entostroma **D** longitudinal sections through pseudostromata **E, F** immature asci **G, H** immaure Ascospores **I, J** conidiophores **K–M** conidia. Scale bars: 1 mm (**A, B**); 0.5 mm (**C, D**); 10 μm (**E–M**).

##### Culture characters.

On PDA at 25 °C, colonies growing slowly and unevenly, reaching 70 mm diam. within 30 d, gradually becoming brownish dark grey in colour with scant cottony aerial mycelium, asexual morphs developed after 40 d.

##### Additional specimen examined.

CHINA. Beijing City: Songshan Mountain, on dead twigs of *Quercusdentata*, 15 June 2018, N. Jiang & C.M. Tian (isotype: BJFC-S1723; living culture from conidium: CFCC 52998).

##### Notes.

So far, ten species and one variety have been described from *Quercus* branches, and they can be distinguished by conidial characteristics (Muthumary and Sutton 1986, Jiang et al. 2018, Table 1). *Coryneumsongshanense* and *C.sinense* can be distinguished from *C.arausiacum*, *C.depressum*, *C.elevatum*, *C.japonicum*, *C.megaspermum*, C.megaspermumvar.cylindricum, *C.neesii*, *C.umbonatum*, and *C.quercinum* by unbranched conidiophores (Sutton 1975, Muthumary and Sutton 1986, Jiang et al. 2018). *Coryneumsongshanense* is obviously distinguished from *C.sinense* in narrower conidia (9–11.5 μm in *Coryneumsongshanense* vs. 13–17 μm in *C.sinense*) and phylogeny (Fig. 1).

## Discussion

In this study, fresh *Coryneum* specimens were collected in China and identified based on combined morphological amd molecular data. Additional accessions of three recently described *Coryneum* species, *C.gigasporum*, *C.sinense*, and *C.suttonii* (Jiang et al. 2018), were identified, with matching conidial characteristics and sequences (Fig. 1). The new species *C.ilicis* was discovered on *Ilexpernyi* (Aquifoliaceae, Sapindales), which represents a new host family and genus for *Coryneum*. *Coryneumcesatii* was reported from the same host order, Sapindales, on branches of *Aesculus* (Hippocastanaceae) (Sutton 1975). The second new species, *Coryneumsongshanense*, was discovered on dead twigs of *Quercusdentata* (Fagaceae, Fagales). Host species belonging to Fagales show higher diversity of *Coryneum* species (Table 1), and it is likely that additional taxa will be discovered by molecular data, considering that in many regions suitable hosts have not yet been adequately studied.

However, most of the *Coryneum* species are lacking DNA sequences, thus species identification based on DNA sequence analyses is presently difficult. Hence, polyphasic approach, i.e. incorporating morphological characters (such as conidial sizes and numbers of distosepta), as well as host associations are important for species identification (Sutton 1975, 1980, Jiang et al. 2018). However, host identifications may be incorrect and many geographical areas remain insufficiently studied. In addition, the morphological characters often significantly overlap between species, which makes identifications solely by morphology challenging. Hence, studies based on the types of already described species and new collections from potential hosts are important to achieve a reliable species classification and circumscription within *Coryneum*.

## Supplementary Material

XML Treatment for Coryneum ilicis

XML Treatment for Coryneum songshanense
